# Gastric cáncer: Overview 

**Published:** 2013-09-30

**Authors:** M. Blanca Piazuelo, Pelayo Correa

**Affiliations:** 1 Division of Gastroenterology, Department of Medicine,Vanderbilt University School of Medicine, Nashville, TN, USA.

**Keywords:** Gastric cancer, gastric adenocarcinoma, *Helicobacter pylori*, epidemiology, multifocal atrophic gastritis, intestinal metaplasia, dysplasia

## Abstract

Gastric cancer ranks fourth in incidence and second in mortality among all cancers worldwide. Despite the decrease in incidence in some regions of the world, gastric cancer continues to present a major clinical challenge due to most cases being diagnosed in advanced stages with poor prognosis and limited treatment options. The development of gastric cancer is a complex and multifactorial process involving a number of etiological factors and multiple genetic and epigenetic alterations. Among the predisposing factors are: *Helicobacter pylori* infection, high salt intake, smoking, and in a small percentage of patients, a familial genetic component. More than 95% of stomach cancer cases are adenocarcinomas, which are classified into two major histologic types: intestinal and diffuse. Intestinal type adenocarcinoma is preceded by a sequence of gastric lesions known as Correa´s cascade and is the histologic type associated with the global decrease in gastric cancer rates. Diffuse type adenocarcinomas have a more aggressive behavior and worse prognosis than those of the intestinal type. According to the anatomical location, adenocarcinomas are classified as proximal (originating in the cardia) and distal (originating in the body and antrum). This classification seems to recognize two different clinical entities. Surgical resection of the tumor at an early stage is the only effective treatment method. Therefore, the identification and surveillance of patients at risk may play a significant role in survival rates. Anti-*Helicobacter pylori* therapy has been shown to be an effective measure in the prevention of gastric cancer.

## Abstract (es)

El cáncer gástrico ocupa el cuarto lugar en incidencia y el segundo en mortalidad entre todos los cánceres en todo el mundo. A pesar de la disminución de la incidencia en algunas regiones del mundo, el cáncer gástrico continúa siendo un reto clínico debido a que la mayoría de los casos se diagnostican en etapas avanzadas con mal pronóstico y opciones de tratamiento limitadas. El desarrollo de cáncer gástrico es un proceso complejo y multifactorial que implica un número de factores etiológicos y múltiples alteraciones. Entre genética y epigenética los factores predisponentes son: infección por Helicobacter pylori, alto consumo de sal, fumar, y en un pequeño porcentaje de los pacientes, un componente genético familiar. Más del 95% de los casos de cáncer de estómago son adenocarcinomas, que se clasifican en dos principales tipos histológicos: intestinal y difuso. Adenocarcinomas de tipo intestinal están precedidos por una secuencia de lesiones gástricas conocidas como cascada de Correa y es el tipo histológico asociado con la disminución global de las tasas de cáncer gástrico. Adenocarcinomas de tipo difuso tienen un comportamiento más agresivo y peor pronóstico que aquellos del tipo intestinal. De acuerdo con la localización anatómica, los adenocarcinomas se clasifican como proximal (originario en el cardias) y distal (que se origina en el cuerpo y antro). Este clasificación parece reconocer dos entidades clínicas diferentes. La resección quirúrgica del tumor en estadios tempranos es el único método de tratamiento efectivo. Por lo tanto, la identificación y vigilancia de los pacientes en situación de riesgo pueden desempeñar un papel importante en las tasas de supervivencia. El tratamiento Anti-Helicobacter pylori ha demostrado ser una medida eficaz en la prevención del cáncer gástrico.

## Introduction

Gastric cancer is one of the most common malignant diseases with one of the highest mortality rates worldwide. Over 95% of gastric cancer cases are adenocarcinomas; therefore, this review will focus on this type of epithelial tumors, with emphases on their epidemiology, pathology and prevention. Adenocarcinomas of the stomach comprise a notably heterogeneous group of malignant lesions with a variety of predisposing conditions and etiologic factors. Other gastric epithelial malignancies include squamous, adenosquamous, undifferentiated carcinomas, medullary carcinoma with lymphoid stroma and neuroendocrine tumors. Non epithelial primary gastric malignancies include lymphomas and mesenchymal tumors^1^.

## Epidemiology

**Figure 1:**
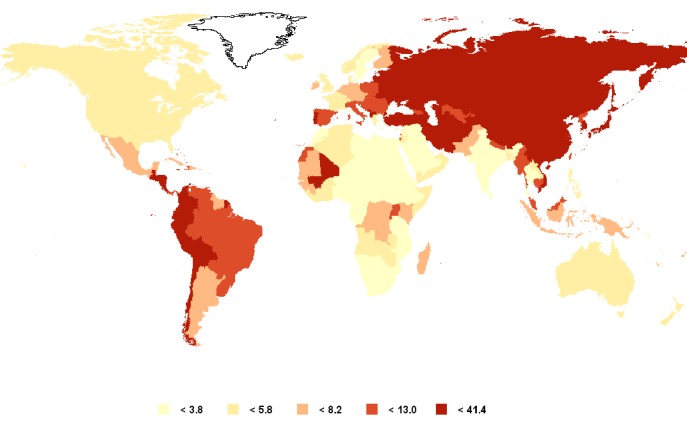
Age-standardized gastric cancer incidence rates, both sexes. GLOBOCAN 2008.3

Gastric cancer ranks fourth in incidence (after lung, breast and colorectal) and second in mortality (after lung cancer) among all cancers worldwide_^2^_.In Colombia, stomach cancer also ranks fourth in incidence (after prostate, breast, and cervical cancers) and second in mortality (after prostate cancer)^3^. It is estimated that in 2008 there were 989,600 new cases and 738,000 deaths from stomach cancer in the world, and approximately 70% of both new cases and deaths occurred in developing countries^2^ ^,^ ^3^. The risk of developing gastric adenocarcinoma increases with age, occurring most frequently between 55 and 80 years of age and it is rare in patients under 30 years. In general, gastric cancer rates are twice as high in men as in women. The highest incidence rates in males are found in Eastern Asia (Korea, Mongolia, Japan, and China with rates between 40 and 60 per 100,000 population), Eastern Europe (around 35 per 100,000), and in some Latin American countries, mainly in Central America and the Andean Region, with rates between 20 to 30 per 100,000 inhabitants. Some of the lowest incidence rates are found in African countries (0.6 to3/100,000) and in North America (5 to6/100,000) (Fig.1).

In Colombia, male and female incidence rates are 23.4 and 12.5 per 100,000 population, respectively. It is important to consider that incidence rates are subject to differences in diagnostic criteria for malignancy among countries. In Japan, the diagnosis of gastric adenocarcinoma is based on nuclear and structural epithelial criteria even without stromal invasion. In contrast, in the Western world, the diagnosis of adenocarcinoma requires the presence of stromal invasion^4^.

There are also significant differences in the risk of gastric cancer in different ethnic groups within a given geographical area. For example, in the United States, Hispanics, African-Americans, and Native Indians are more affected than Caucasians. However, these differences cannot be generalized as simple racial predisposing differences as low socioeconomic status is also associated with increased gastric cancer risk. There is also an important environmental component since a decrease in gastric cancer risk has been described in populations that migrated from a high incidence to a low incidence geographical region.

During the past 50 years, the incidence rates of stomach cancer have been declining steadily in many parts of the world. It is believed that this decrease is largely due to factors associated with the use of refrigerated foods, the availability of fresh fruits and vegetables, and the decrease in the use of salt as a means of preserving food. Other probably associated factors include the decrease in the prevalence of *Helicobacter pylori* infection in many countries and the decline in smoking in some industrialized countries^2^.

In spite of the decreasing incidence of gastric cancer in many countries, the last four decades have seen an increase in cases of cancer arising in the cardia, accompanying an increase in the incidence of adenocarcinomas at the esophagogastric junction and at the distal esophagus, especially in male, white patients. This increase has been observed in some European countries, in the United States^5^ ^,^ ^6^ and also in Colombia^7^. In the United States, the rates of adenocarcinoma of the cardia have stabilized over the past decade. Another phenomenon, recently identified in the United States where the overall incidence rate has been declining, has been the increase in the rate of distal gastric cancer in Caucasians of both sexes in the 25-39 year old age group. This increase has been present during the past three decades and its causes are still unknown.

## Etiology

Gastric cancer is a multifactorial disease. There is a variety of environmental, infectious, and host-related factors that may interact favoring the development of the disease. Some worthy of mention are: *H.pylori*. In 1994 the International Agency for Research on Cancer recognized the *H.pylori* infection as a type I carcinogen^8^. It has been estimated that the infection with this bacterium is responsible for 77% of the cases of distal gastric cancer and is associated with both types, intestinal and diffuse, but not with carcinomas originating in the gastric cardia^9^.* Helicobacter pylori* is also recognized as an etiologic agent in lymphomas originating in the mucosa-associated lymphoid tissue (MALT lymphomas).

*Helicobacter pylori* is a Gram-negative spiral bacterium with a wide variety of mechanisms that allow for colonization of the gastric mucosa and evasion or modification of the host´s immune response^10^. The infection is usually acquired in childhood and can persist for decades unless treatment and eradication of the bacteria is accomplished. No one knows exactly what the transmission mechanism is, but it is believed to be acquired orally from person to person. Infection of the gastric mucosa by *H.pylori* induces an acute and chronic inflammatory reaction whose intensity depends on factors associated with the host and the bacterium. This prolonged inflammatory reaction, or active chronic gastritis, is believed to be one of the main factors contributing to the malignant transformation of the epithelium.

Multiple mechanisms have been described for carcinogenesis associated with inflammation caused by *H.pylori*. Among them, it is important to note the production of reactive oxygen species that lead to oxidative DNA damage and mutations, aberrant DNA methylation (mainly hypermethylation of CpG islands in the promoter regions of certain genes) that leads to silencing of tumor suppressor genes. In one of our studies in Colombian with patients from the province of Nariño, significantly greater methylation levels of *RPRM* (reprimo, a tumor suppressor gene) were found in the gastric mucosa of subjects living in an area of high risk for gastric cancer (Túquerres) than in one of low risk (Tumaco). In addition, significantly elevated methylation levels were found in patients infected with the virulent *cagA*-positive *vacA* s1/m1 *H.pylori* strains^11^.

There is great genetic variety among *H.pylori* strains, with variable pathogenic and carcinogenic potential, and a given individual may be infected simultaneously with multiple strains. *H.pylori* is equipped with a number of adhesins which allow them to adhere to the gastric epithelium. This adhesion is essential for ensuring the prolonged persistence of the bacterium in the stomach and for the injection of CagA and other proteins in to epithelial cells, using a type IV secretion system. CagA is an oncogenic protein, a product of the bacterial gene *cagA*. This gene is part of a DNA region called the *cag* pathogenicity island (*cag*PAI), which is not present in all strains of *H.pylori* and is considered a virulence marker^12^. Infection with *cag*-positive strains is associated with a greater risk of severe gastritis, premalignant gastric lesions,and gastric cancer than is infection with *cag*-negative strains^13^. Almost all East Asian strains are *cag*-positive, while 60-70% are *cag*-positive amongs trains of the Western world. In Colombia, it is estimated that 80% of the adult population is infected with *H. pylori* and that 70-80% of the strains obtained from symptomatic patients are *cag*-positive^14^ ^,^ ^15^. A greater prevalence of *cag*-positive strains has been observed in regions with a greater incidence of gastric cancer^14^.

After entering the epithelial cells, CagA is phosphorylated in motifs containing the aminoacid sequence EPIYA, which triggers a series of events that leads to morphological epithelial alterations^12^. These EPIYA motifs are classified into types A, B, C or D, according to the amino acids flanking the EPIYA sequence. The number and type of EPIYA motifs varies among strains of *H.pylori*. In Western countries, the strains have EPIYA types A, B, and C. In East Asian countries, strains contain type D sequences instead of type C. The presence of more than three EPIYA sequences is a factor that significantly increases the risk of gastric atrophy, intestinal metaplasia and gastric cancer^12^ ^,^ ^16^. *In vivo* and *in vitro* studies have shown that *CagA* induces ruptures of the epithelial intercellular junctions, loss of epithelial polarity, increased cell proliferation, reduced apoptosis, and development of adenocarcinomas^12^.

Another *H. pylori* gene associated with virulence is *vacA*, which encodes a bacterial toxin(VacA) that induces cytoplasmic vacuoles, pores in the mitochondrial membranes and apoptosis of epithelial cells. Although all *H.pylori* strains possess the *vacA* gene, genetic variations determine the functional activity of *VacA* and the risk of gastric disease^17^. The regions of greatest genetic variation are: the s(signal) region with alleles s1a, slb, slc, or s2; the m(middle) region with m1 or m2 alleles; and the i(intermediate) region with type i1 or i2 alleles. *vacA* s1/m1 or *vacA* s1/m1/i1 strains are more associated with an increased risk of progression of premalignant lesions and with gastric cancer than are *vacA* s2/m2 or *vacA* s2/m2/i2 strains^12^.

Additionally, certain adhesion proteins present on the outer membrane of some *H.pylori* strains are associated with virulence. BabA (blood-group antigen-binding adhesion) is encoded by *babA2*, a gene not found in all strains. BabA adheres to the Lewis blood group antigen present on the membrane of gastric epithelial cells. Infection with *babA2*-positive strains is associated with increased risk of gastric adenocarcinoma^12^.

The association between the prevalence of *H. pylori* infection and the risk of gastric cancer seems to be challenged in some parts of the world. In some African countries, the prevalence of * H. pylori* infection is very high but the risk of gastric cancer is very low, a phenomenon called the African enigma^18^. This phenomenon has been described in other parts of the world and has stimulated numerous epidemiological studies aimed at finding its´ causes. In addition, despite its relatively recent implication as an etiological agent of gastric cancer and its broad worldwide distribution, less than 1% of persons infected with *H. pylori* will develop stomach cancer. *H. pylori * has been part of the human microbiome from ancient times. Both species (*Homo sapiens* and *H.pylori*) migrated together from Africa about 60,000 years ago and spread to the other continents^19^ ^,^ ^20^. *Helicobacter pylori* strains display an extraordinary genetic diversity, mainly due to high rates of mutation and genetic recombination. Based on the study of gene sequences, a growing variety of phylogenetic groups and subgroups of *H. pylori* have been described over the past decade that reflect human migrations: hpEurope, hpAfrica1 (including hspWAfrica and hspSAfrica), hpAfrica2, hpEastAsia (including hspAmerind, hspEAsia and hspMaori), hpAsia2 and hpSahul^21^.

In one of our studies in Colombia, *H.pylori* strains and gastric histopathology of 80 adult patients with dyspeptic symptoms were analyzed^22^. The study included residents of Túquerres, in the Andean Mountains, where the risk of gastric cancer is very high, and of Tumaco, on the Pacific coast, where the risk is much lower. Of a total of 64 *cagA*-positive *vacA* s1m1 *H. pylori* strains, all strains from the mountainous area (n=35), where the population is mainly mestizo (mixed Amerindian and European origin), were classified as hpEurope, indicating the exposure of Amerindian strains to those of European origin. In contrast, among the 29 *cagA*-positive *vacA* s1m1 strains obtained from the inhabitants of Tumaco (of mixed African and European ancestry), 10 were classified as hpEurope and 19 as hpAfrica1^22^. Regardless of the place of residence, patients infected with European strains have significantly more advanced precancerous histological lesions and greater oxidative damage in the gastric mucosa than did those infected with strains of African origin. These findings suggest that among *cagA*-positive *vacA* s1m1 strains, considered virulent in general, the phylogenetic classification may be a determining factor in the risk of progression to stomach cancer. African strains seemed to be less virulent than strains of European origin.

Results of this and other studies indicate that European and African immigrants brought their own *H.pylori * strains, which have been preserved over the centuries, extensively replacing Amerindian strains from the native people of America. It is likely that some combinations of *H.pylori* and *Homo sapiens* of different ancestral origins lead to interactions that may modify gastric cancer risk.

### Epstein-Barr virus (EBV).

Multiple studies in different parts of the world have found the presence of EBV in 5 to 16% of gastric carcinomas, which supports its possible role as an etiologic agent^23^ ^,^ ^24^. A meta-analysis of 70 studies found a pooled prevalence of EBV positivity of 9% in gastric cancer cases. This prevalence was similar in cases from Asia, Europe, and the Americas.^25^Male patients were twice as likely to have EBV-positive tumors as female patients, and tumors arising in the gastric cardia or corpus were more than twice as likely to be EBV-positive as those in the antrum. Tumors in post surgical gastric stump/remnants had a prevalence of EBV four times higher than primary non-remnant tumors. No difference in EBV prevalence between intestinal and diffuse histologic types was found, and a strong association (>90%) of EBV with the uncommon lymphoepithelioma-like carcinoma was confirmed^25^. A recent multicentric study suggests that EBV positivity is a favorable prognostic indicator of survival^26^. One of the strongest observations that support the role of EBV as a causative agent is the clonal presence of the virus uniformly distributed in all malignant cells of EBV-positive tumors. This is not observed in the non-tumoral epithelial cells that surround the tumor.^23^ However, the role of EBV in gastric carcinogenesis is not yet clearly defined.

### Environmental and lifestyle factors.

Tobacco smoking is a recognized risk factor for the development of gastritis, ulcers, intestinal metaplasia, and both proximal and distal gastric cancer. It is estimated that up to 18% of stomach cancer cases are attributable to tobacco smoking^27^and there is evidence supporting a positive interaction between tobacco smoking and *H. pylori* infection.

Among dietary factors, high salt intake is associated with an increased risk of gastric cancer. A proposed mechanism is the direct damage to the gastric mucosa with subsequent inflammatory response and increased cell proliferation. Additionally, in the presence of *H.pylori* infection, high salt intake further increases the risk of gastric cancer. As a mechanism associated with this interaction, *in vitro* studies have shown increased expression of the oncogenic protein CagA when *H. pylori* is grown in media with high salt concentrations. Analysis of 36 *cagA*-positive *H. pylori* strains from Colombian patients demonstrated considerable heterogeneity in salt-regulated CagA expression. These differences are attributable to variation in a DNA motif in the *cagA* promoter region.^28^Host iron levels have also been associated with gastric cancer risk. In a Colombian population at high risk of gastric cancer, significantly lower serum ferritin levels were found in subjects with advanced gastric precancerous lesions when compared with subjects with non-atropic gastritis^29^. In addition, *H. pylori* isolates from subjects with the lowest ferritin levels showed greater virulence potential *in vitro* than isolates from patients with the highest ferritin levels^29^.

Meat consumption is a possible risk factor for gastric cancer. Some studies have found a significant positive association between processed meat consumption and gastric cancer. A large scale European study found a significant association between meat consumption and distal gastric cancer; this association was stronger in subjects infected with *H. pylori* ^30^. With regard to alcohol consumption, results have been inconsistent, although a recent meta-analysis found positive association between heavy alcohol drinking and gastric cancer risk.^30^A factor associated with decreased risk of gastric cancer is the adequate intake of fresh fruits and vegetables^31^. It is thought that the protective mechanism is related to the antioxidant capacity of these foods.

## Host Factors

### Genetic polymorphisms.

A great variety of genetic polymorphisms have been associated with gastric cancer risk, mainly in some inflammation-related genes, such as *IL1B*, *IL1RN*, *IL10* and *TNF*. Interleukin (IL)-1β and tumor necrosis factor (TNF)-α are potent pro-inflammatory cytokines with suppressive properties for the production of gastric acid. IL-10 is an anti-inflammatory cytokine that counteracts the effects of pro-inflammatory cytokines, and variants have been identified in *IL10* that influence its production. The colonization of the gastric mucosa by *H. pylori * elicits an immune response that includes a wide variety of pro- and anti-inflammatory cytokines. It is believed that the gastric acid suppression caused by IL-1β and TNF-α favors the spread of *H.pylori* from the antrum to the gastric body and fundus, leading to a more extensive and severe gastritis that results in gastric atrophy and subsequently in cancer. In addition, an animal model has demonstrated that elevated levels of IL-1β are sufficient to induce dysplasia and gastric cancer^32^. Subjects carrying the *IL1B-511T* allele are high producers of IL-1β^33^, and multiple studies confirm this polymorphism as a risk factor for intestinal-type gastric adenocarcinoma in Caucasian populations (or in non-Asian populations) but not in Asian populations^34^ ^,^ ^35^. Carriers of *IL1RN2* of non-Asian origin have shown an increased risk of gastric adenocarcinoma (intestinal and diffuse types) predominantly for distal tumors^35^. Also associated with increased risk of gastric cancer are: the allele *TNF-308A* in Caucasian populations and the *IL8-251AA* genotype in Asian populations. A reduced risk of gastric cancer has been found in carriers of *IL1B-31C* of Asian origin and in carriers of *IL10-592A* (including all ethnic groups).

Genome-wide association studies, a powerful new approach to identifying susceptibility loci, are used to analyze hundreds of thousands of single nucleotide polymorphisms simultaneously in large populations. Several gastric cancer susceptibility loci have been identified opening new doors in cancer research^36^.

### Familial gastric cancer.

The risk of developing gastric cancer is two to ten times greater in subjects with a family history^30^. Most familial cases are considered sporadic and seem to be influenced by shared environmental factors, such as *H.pylori* infection, diet, and socioeconomic status. However, gastric cancer can develop as part of familial cancer syndromes including hereditary diffuse gastric cancer syndrome, Lynch syndrome, familial adenomatous polyposis, Peutz-Jeghers syndrome and Li-Fraumeni syndrome^37^. Hereditary diffuse gastric cancer is a rare, autosomal dominant disorder that causes 1-3% of cases of familial gastric cancer cases. This syndrome is caused by a variety of mutations of the gene encoding E-cadherin, a cell adhesion protein essential for the maintenance of epithelial tissue architecture^38^. In the presence of one of these mutations, the life time risk of developing gastric cancer is 70% to 80%. Therefore, a prophylactic gastrectomy is recommended around 20 years of age.

### Other conditions:

Pernicious anemia, Menetrier's disease and gastric stumps are other conditions that increase the risk of gastric cancer.

## Classification Systems

Gastric adenocarcinomas are classified into two main groups: early and advanced, which are associated with prognosis. The 5-year survival rate in patients with early gastric cancer is between 85 and 100%, while it is only 5-20% for advanced gastric cancer. Early gastric carcinoma is defined as limited to the mucosa or sub-mucosa, regardless of lymph node invasion^39^. Advanced gastric cancer is classified based on the gross appearance into four types: I) polypoid, II) ulcerated with well-defined borders, III) ulcerated with irregular and infiltrative borders, and IV) diffuse infiltrative, with no evidence of mass or ulcer^39^. The last type is usually observed in diffuse type adenocarcinomas and it is known as *linitis plastica*.

According to their anatomical location, gastric adenocarcinomas are classified as proximal (originating in the cardia) and distal (originating distal to the cardia). The gastric cardia is an ill-defined region, 0.1 to 1cm in length, located immediately distal to the squamocolumnar junction. Tumors involving the squamocolumnar junction are frequently discovered in advanced stages when the size of the tumor makes it difficult to properly assess the site of origin as the esophagus or stomach. Previously, it was left to the judgment of the physicians involved to classify the tumors located at the esophago gastric junction as esophageal or gastric. Currently, a new approach is followed due to the recognition of proximal and distal adenocarcinomas as two distinct biological entities with different clinical features and epidemiological characteristics. Unlike distal adenocarcinomas, cardia adenocarcinomas have shown increased rates in the last several decades, especially in industrialized countries, affect predominantly Caucasian populations, and are associated with gastroesophageal reflux disease but not with *H.pylori* infection. In addition, cardia adenocarcinomas seem to have a more aggressive natural history with deeper gastric wall invasion, greater lymph node involvement, and a worse prognosis than distal adenocarcinomas^5^ ^,^ ^40^. In the United States, the five-year survival rate for proximal and distal carcinomas is 14% and 26%, respectively^5^. Based on these observations and due to the similar characteristics shared by esophageal and esophagogastric adenocarcinomas, the 7th edition (2009) of the American Joint Classification of Cancer(AJCC) Tumor Staging Manual, uses the esophageal cancer staging system for all cancers arising in the esophagogastric junction and any cancer arising in the proximal 5 cm of the stomach with involvement of the esophagogastric junction^41^.

## Histological Classification

There are several histological classification systems, but the most frequently used worldwide is the Laurén classification^42^. Other widely used systems include that of the World Health Organization (WHO)^1^ and the classification of the Japanese Gastric Cancer Association^39^. The Laurén classification recognizes two main histological types: intestinal and diffuse, which present differences in clinical and epidemiological characteristics. A small percentage of adenocarcinomas are mixed, presenting features of both types.

### The precancerous cascade.

**Figure 2:**
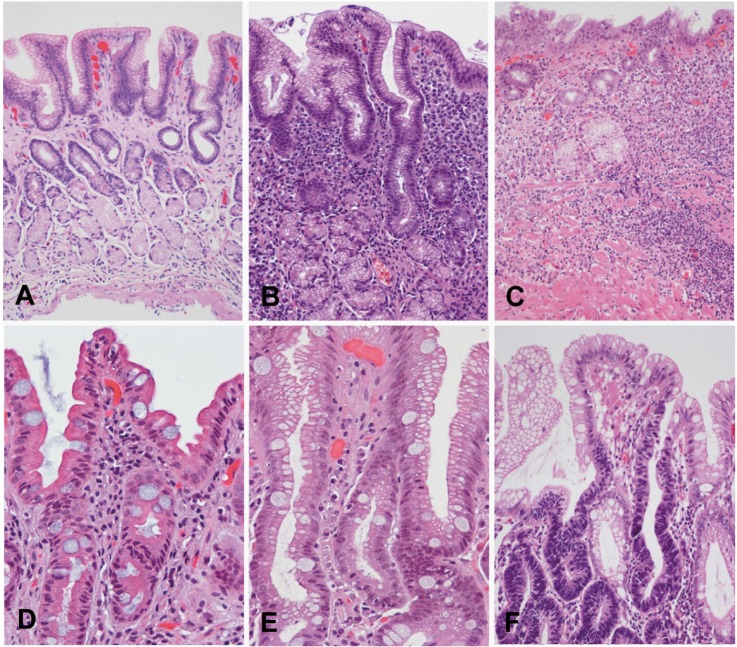
Correa's precancerous cascade. A, Normal gastric mucosa. B, Non atrophic chronic gastritis. Abundant inflammatory infiltrate in lamina propria with well-preserved glands observed in the deeper half of the mucosa. C, Multifocal atrophic gastritis without intestinal metaplasia. Marked loss of glands, with prominent inflammatory infiltrate and proliferation of fibrous tissue in the lamina propria. D,Intestinal metaplasia, complete type. Goblet cells alternating with absorptive enterocytes that present well-developed brush border. E, Intestinal metaplasia, incomplete type. Goblet cells alternating with columnar cells that contain mucin droplets of variable sizes. F, Dysplasia. Epithelium with high-grade dysplasia (lower half of the photograph) occurring in a background of incomplete metaplasia (observed in the foveolar superficial epithelium). (H&E; original magnification: A-C x100; D-F x200).Images A-Care reproduced with permission reference 60.

The intestinal type of gastric adenocarcinoma is preceded by a sequence of histological lesions (known as Correa´s cascade) with well-defined characteristics: non-atrophic gastritis → multifocal atrophic gastritis without metaplasia → intestinal metaplasia of the complete type → intestinal metaplasia of the incomplete type→ dysplasia^43^ ^,^ ^44^ (Fig. 2). This precancerous process was described in 1975 by Correa *et al.* based on observations in Colombian populations at high risk of gastric cancer^45^ ^-^ ^47^. Following the identification of *H. pylori * as a causative agent of gastritis in 1983^48^, it was recognized that this process is initiated and sustained by the infection with this bacterium and may last for decades preceding the malignant transformation.

Although *H. pylori* remain primarily in the gastric lumen, the interaction with the epithelium causes an inflammatory reaction that promotes the migration of inflammatory cells to the mucosa. This immune response is not effective in eliminating the bacterium and may last years to decades, causing chronic active gastritis, unless the infection is eradicated. Prolonged and severe gastric inflammation may cause epithelial damage, which may lead to gastric atrophy, characterized by loss of parietal and chief cells in the oxyntic mucosa and/or loss of glandular units in the oxyntic or antropyloric mucosa. Glandular atrophy may occur simultaneously in multiple isolated foci distributed throughout the gastric mucosa; therefore, it is known as multifocal atrophic gastritis. Subsequently, the gastric epithelium may be replaced by epithelium with intestinal phenotype. Intestinal metaplasia arises most often in the lesser curvature of the stomach, but may be observed anywhere in the stomach. In the oxyntic mucosa, parietal and chief cells may be replaced by mucous cells (pseudopyloric metaplasia). Both intestinal and pseudopyloric metaplasias are manifestations of gastric atrophy. Over time, the foci of atrophy and metaplasia may increase in size, coalesce, and occupy large areas. Two main types of intestinal metaplasia are recognized: complete (small intestine type, type I) and incomplete (colonic, including types II and III) with different risks of progression to cancer.

Complete intestinal metaplasia is characterized by well-developed goblet cells alternating with mature absorptive cells. Paneth cells may also be observed. Incomplete metaplasia consists of goblet cells alternating with columnar mucous cells containing varying amounts of intracytoplasmic mucin. Long-term observations support the hypothesis that complete intestinal metaplasia occurs initially and may transform over time into incomplete metaplasia. The former carries a lower risk of gastric cancer, whereas the latter presents a much higher risk^49^. Eventually, foci of dysplasia may arise in metaplastic epithelium; these foci are usually small and therefore the diagnosis is subject to sampling error. Dysplasia (glandular intraepithelial neoplasia or non-invasive neoplasia) is a neoplastic lesion limited to the epithelium, without invasion of the lamina propria, and is classified mainly as low grade and high grade. Low-grade dysplasia is characterized by crowded glands lined by columnar cells with hyperchromatic, elongated, and pseudo-stratified nuclei that maintain polarity with respect to the basement membrane. Low-grade dysplasia shows minimal glandular architectural alteration and the cells show mild to moderate atypia. In high-grade dysplasia, dysplastic cells are usually cuboidal rather than columnar, with a high nuclear-cytoplasmic ratio, loss of nuclear polarity, and prominent nucleoli. There is prominent mitotic activity, sometimes with atypical mitoses, and marked architectural distortion with glandular crowding, branching and budding. In Japan, high-grade dysplasias are classified as intramucosal carcinomas^4^. The WHO recommends the terms low-grade and high-grade intraepithelial neoplasia/dysplasia, and defines carcinoma as the invasion of the lamina propria or beyond.

### The intestinal type adenocarcinoma

**Figure 3:**
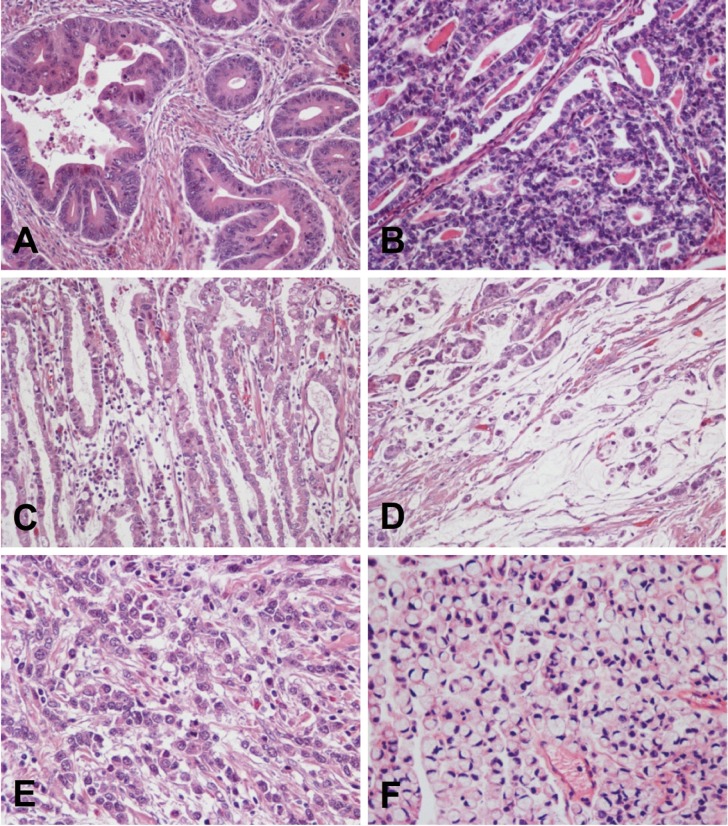
Gastric adenocarcinoma. A-C, Intestinal type. Three different tumors are shown with formation of irregular glands, tubules and papillae. D, Mucinous adenocarcinoma, with small groups of tumor cells floating in pools of mucin. E y F, Diffuse type. Two different tumors are shown composed of non cohesive individual cells infiltrating the stroma. Signet ring cell carcinoma (F) is formed by cells with abundant intracytoplasmic mucin and nuclei displaced to the periphery. This morphology is characteristic of this tumor type.

Is thought to originate from the precancerous lesions previously described. It is the histological type that predominates in high risk areas of gastric cancer and the type associated with the global decrease in gastric cancer incidence. It usually occurs in individuals between 55 and 80 years of age, and the male-to-female ratio is 2:1. Histologically, it is characterized by malignant epithelial cells that show cohesiveness and glandular differentiation infiltrating the stroma. Tumor cells may display varying degrees of nuclear atypia and may present tubular, trabecular, papillary or tubulo-papillary structures (Fig.3A-C). According to cellular and architectural criteria, gastric adenocarcinomas are classified as well, moderately and poorly diferentiate^1^.

### The diffuse type adenocarcinoma

Occurs in younger patients (40-60 years of age); there is on male sex predominance, and it generally carries a worse prognosis than the intestinal type. This tumor is composed of discohesive cells that infiltrate the stroma individually or in small groups (Fig.3E-F). In some cases the tumor may form solid masses, but even in these cases the cells appear to be loose, with little cohesiveness and do not form epithelial cords. Occasionally, tiny glandular lumens can be observed, formed by cells that have no polarity and are surrounded by a diffuse infiltration of isolated tumor cells^42^. A variant of the diffuse histological type is the signet ring cell adenocarcinoma. By definition, it must be predominantly composed (>50%) of signet ring cells^1^. These cells are characterized by abundant cytoplasmic mucin that displaces the nucleus to the periphery (Fig.3F).

Mucinous (colloid) adenocarcinomas may originate in adenocarcinomas of either intestinal or diffuse types^42^. These tumors are composed of malignant epithelial cells floating loose or in small groups in large mucinous areas (Fig. 3D); in some cases, signet ring cells may be observed. By definition, the extracellular mucinous pools constitute at least 50% of the tumor^1^.

## Prevention and early detection

General measures for gastric cancer prevention are the adequate consumption of fresh fruits and vegetables, the avoidance of excessive salt intake and the avoidance of exposure to tobacco smoke. Treatment and eradication of *H. pylori* infection is recommended in patients with gastritis, and there are a number of studies showing the benefit of anti-*H.pylori* treatment in reducing both the progression of precancerous lesions and the risk of gastric cancer^50^ ^-^ ^52^. Large-scale *H. pylori* eradication as a preventive measure for gastric cancer has been the object of controversy. This measure is not recommended due to the risk of the appearance of antimicrobial-resistant strains, not only for *H. pylori* but for other infectious agents as well. Furthermore, although the efficacy of treatment regimens have shown recent improvements, virtually all schemes present some margin of failure in eradicating the bacteria and the rates of re-infection are often high. Moreover, since *H.pylori* has accompanied human beings from ancient times, strong arguments exist that consider this bacterium as part of the human gastric microbiome and its elimination as the cause of the increase in prevalence of diseases such as gastroesophageal reflux, Barrett's esophagus, and esophageal and cardia adenocarcinoma^10^.

Because gastric cancer develops through a long period of precancerous lesions that can take years to decades, the identification of these lesions and endoscopic surveillance of patients at high risk of gastric cancer may help to detect early-stage malignant lesions when they are still operable and have high probability of cure. A large scale study estimated that the progression rates to gastric cancer within 10 years of follow-up for patients with atrophic gastritis, intestinal metaplasia, low-grade dysplasia or high-grade dysplasia were 0.8%, 1.8%, 4% and 33%, respectively^53^. It is recommended that patients undergoing upper gastrointestinal endoscopy due to gastrointestinal symptoms have a sampling of at least five biopsies, including two of gastric antropyloric mucosa (greater and lesser curvature), one of the *incisura angularis*, and two of the gastric body (lesser and greater curvature). If*H.pylori* infection is diagnosed, treatment leading to the eradication of the bacteria should be given. The premalignant gastric lesion most frequently identified is intestinal metaplasia, but the vast majority of patients with intestinal metaplasia never develop gastric cancer. As mentioned, the incomplete type of intestinal metaplasia has a greater risk for gastric cancer than the complete type^49^ and the extent of intestinal metaplasia is directly proportional to the risk of gastric cancer. Although there are not clearly defined criteria, intestinal metaplasia may be considered extensive when it is present in more than one of the biopsy samples obtained from different regions of the stomach during an endoscopic procedure, or when the metaplasia occupies a large part of a gastric biopsy. We proposed in 2010 an algorithm for monitoring patients with intestinal metaplasia^54^. Individuals with extensive or incomplete intestinal metaplasia obtained during an endoscopic procedure that did not follow antrum and corpus mapping should undergo a new endoscopic procedure with mapping within a year, and afterwards every three years if the lesion persists. However, the management should be individualized according to other risk factors, such as family history of gastric cancer, geographic origin, smoking, or persistence of gastrointestinal symptoms.

In order to unify the histopathological evaluation of atrophic gastric lesions an international group of pathologists proposed a system (Operative Link for Gastritis Assessment, OLGA) based on stages associated with cancer risk (O-IV)^55^. Using a protocol based on five gastric biopsies and using detailed visual scales (that include atrophy without metaplasia, intestinal metaplasia and pseudopyloric metaplasia), it is proposed that the system be applied consistently and could be used in the routine practice of histopatology to detect subjects at gastric cancer risk^55^. Subsequently, another system based only on the evaluation of the extent of intestinal metaplasia (OLGIM) was proposed^56^, which does not recognize gastric atrophy without metaplasia or pseudopyloric metaplasia as markers of gastric cancer risk. Both systems ignore the type of intestinal metaplasia as a risk factor, although a later study reported a strong association between intestinal metaplasia of the incomplete type and the high-risk OLGA stages^57^.

A recent consensus in Europe recommended taking "at least four stomach biopsies, proximal and distal, of the lesser and greater curvatures" for proper evaluation of premalignant gastric lesions, without emphasizing sampling of the *incisura angularis* that we consider indispensable. This consensus recommended that patients with atrophy and/or extensive intestinal metaplasia should be followed-up with endoscopye very three years and that those with mild to moderate atrophy and/or intestinal metaplasia limited to the antrum do not need follow-up^58^. The presence of dysplasia is an indicator of high gastric cancer risk and it should be confirmed and classified by two gastrointestinal pathologists, due to the high inter-observer variability. According to the noted recent consensus, in patients with dysplasia and no endoscopically visible lesion, if the dysplasia is of high-grade it must be followed-up immediately (with endoscopy and mapping) and between 6 and 12 months later, and if the dysplasia is of low grade, it should be followed-up within the following 12 months^58^. If there is dysplasia with a visible lesion at endoscopy, it should be classified according to stage and resection of the lesion must be carried out^58^.

In Japan and Korea, where gastric cancer is highly prevalent, national programs for mass screening and early detection have been implemented. Case-control studies from Japan show a 40-60% decrease in mortality from gastric cancer in those who have been screened. However, data from prospective series that defined death from gastric cancer as an endpoint are inconsistent^59^. There is a great need for non-invasive screening tests that can be applied to large populations to assess cancer risk or detect early gastric cancer cases. Serum pepsinogen (PG) levels have shown great potential for establishing the extent of gastric atrophy in some populations. Serum pepsinogen I is secreted in the oxyntic mucosa, and PGII is secreted in both oxyntic and antropyloric mucosa. In presence of atrophy of the oxyntic mucosa, there may be a decrease in both PGI and PGII, but PGI usually shows a more marked decrease. In Japan, severe gastric atrophy is diagnosed when serum PGI levels are <70 µg/L and a PGI/PGII ratio <3; these levels have proven useful for the identification of subjects at high risk, for whom follow-up should be offered^59^. Studies outside of Japan have shown inconsistent results and serum PG levels are not widely used for the identification of subjects at risk. Other serological markers for the detection of precancerous gastric lesions or early gastric cancer, such as trefoil factor 3, hypermethylation of genes and microRNAs,have been proposed and are subject of intense investigation.
